# Follistatin‐like protein 1 (FSTL1) promotes chondrocyte expression of matrix metalloproteinase and inflammatory factors via the NF‐κB pathway

**DOI:** 10.1111/jcmm.14155

**Published:** 2019-01-15

**Authors:** Peng‐Fei Hu, Chi‐Yuan Ma, Fang‐Fang Sun, Wei‐Ping Chen, Li‐Dong Wu

**Affiliations:** ^1^ Department of Orthopedic Surgery, School of Medicine Second Affiliated Hospital, Zhejiang University Hangzhou Zhejiang P.R. China; ^2^ Orthopedics Research Institute of Zhejiang University Hangzhou Zhejiang P.R. China; ^3^ Key Laboratory of Cancer Prevention and Intervention, School of Medicine, China National Ministry of Education, The Second Affiliated Hospital, Cancer Institute Zhejiang University Hangzhou Zhejiang P.R. China

**Keywords:** chondrocyte, follistatin‐like protein 1, interleukin‐1β, matrix metalloproteinase, nuclear factor kappa B, osteoarthritis

## Abstract

**Background:**

The expression of follistatin‐like protein 1 (FSTL1) is closely associated with diseases of the musculoskeletal system. However, despite being a well characterized inflammatory mediator, the effects of FSTL1 on chondrocytes are not completely understood. In this study, we investigated the effects of FSTL1 on the expression of inflammatory and catabolic factors in rat chondrocytes.

**Methods:**

Rat chondrocytes were treated directly with various concentrations of FSTL1 in vitro. The levels of matrix metalloproteinases (MMPs), inducible nitric oxide synthase (iNOS), cyclooxygenase (COX)‐2, interleukin (IL)‐1β, tumour necrosis factor (TNF)‐α and IL‐6 were measured by polymerase chain reaction, ELISA and Western blotting. In addition, activation of the nuclear factor kappa B (NF‐κB) pathway was explored to identify potential regulatory mechanisms.

**Results:**

Follistatin‐like protein 1 directly increased the expression of MMP‐1, MMP‐13, iNOS, COX‐2, IL‐1β, TNF‐α and IL‐6 at both gene and protein level in a dose‐dependent manner. Activation of NF‐ κB and phosphorylation of p65 were also promoted by FSTL1 stimulation.

**Conclusions:**

Follistatin‐like protein 1 exerts pro‐inflammatory and catabolic effects on cultured chondrocytes via activation of the NF‐κB signalling pathway. FSTL1 may therefore be a target in the treatment of OA.

## 1 INTRODUCTION

Osteoarthritis (OA) is a chronic, progressive, debilitating disease and a major cause of disability in the elderly. As a disease affecting the whole joint, OA is characterized by the loss of articular cartilage, synovial inflammation and subchondral bone alteration.^1^ However, as the mechanism giving rise to OA is still unknown, disease‐modifying treatment is unavailable. Current therapeutic approaches, including non‐operative treatments such as physiotherapy, oral drugs and intra‐articular injection, partly improve symptoms, mobility and function.^2^, ^3^ However, considering the potential side effects and short‐term pain relief conferred by medications, the need for a structure‐modifying treatment for OA remains.

Catabolic and inflammatory mediators are associated with the development of OA and have been targeted in drug studies. Diacerein, an inhibitor of IL‐1β in vitro, was shown to be an effective treatment for patients with symptoms of OA.^4^ Tanezumab, a newly developed monoclonal antibody against nerve growth factor, relieves pain and stiffness in patients with moderate‐to‐severe OA of the knee.^5^ Thus, there is increasing interest in new therapies targeting OA.

Follistatin‐like protein 1 (FSTL1), a secreted glycoprotein, was first identified by Shibanuma et al in 1993, as a transforming growth factor (TGF)‐β‐inducible protein.^6^ Previous studies indicated that FSTL1 is involved in the progression of several diseases, including pulmonary fibrosis, cardiovascular disease, cancer and arthritis.^7^, ^8^, ^9^, ^10^, ^11^ In the past decade, the importance of FSTL1 in the pathogenesis of arthritis has been determined, beginning with a study showing that anti‐FRP antibody levels are higher in the synovial fluid and serum of rheumatoid arthritis (RA) patients than in patients with other systemic rheumatoid diseases.^12^ The same group of researchers then reported that transformation of the TGF‐β gene into synovial cells enhanced FSTL1 gene expression. And that FSTL1 protects against the joint erosion seen in RA by down‐regulating the production of matrix metalloproteinase (MMP)‐1, MMP‐3 and prostaglandin E2.^13^ Yury et al found a significant positive correlation between serum FSTL‐1 levels and the arthritis index in mice with collagen‐induced arthritis. The authors also reported that FSTL1 directly increases the expression of pro‐inflammatory cytokines, such as interleukin (IL)‐6 and MCP‐1.^10^ A recent study revealed that human recombinant FSTL1 increased the expression of MMP1, MMP3 and MMP1 via the NF‐κB and mitogen‐activated protein kinase (MAPK) signalling pathway in fibroblast‐like synoviocytes.^14^ Taken together, these results suggest that FSTL1 accelerates RA progression by stimulating inflammation and catabolism. Moreover, FSTL1 expression was shown to be higher in the synovial tissues of OA patients. The same study demonstrated the utility of serum FSTL1 levels as a biomarker of the severity of cartilage destruction.^15^

Despite the evidence linking FSTL1 to RA and OA, its exact role in chondrocytes is not fully understood. Thus, the aim of this study was to explore the effects of FSTL1 on cultured rat chondrocytes. The levels of inflammatory and catabolic mediators induced by different concentrations of FSTL1 were examined, together with the effect of FSTL1 on the nuclear factor kappa B (NF‐kB) pathway.

## 2 MATERIALS AND METHODS

### 2.1 Cells culture and treatment

A total of 18 rats (male:female, 1:1) were housed at 25˚C with 45%‐75% relative humidity and enough food and water (12 hours light/dark cycle). After killed by intraperitoneal injection with 10% chloral hydrate (4 mL/kg; Sigma Aldrich; Merck KGaA, Darmstadt, Germany), the articular cartilage was removed from the tibial plateau and femoral condyle and minced into 1‐3 mm^3^ pieces. After digestion of the tissue using pronase and collagenase, the resulting cells were isolated by centrifugation and cultured as a monolayer in complete fresh Dulbecco's modified Eagle's medium (DMEM; Sigma‐Aldrich; Merck KGaA, Darmstadt, Germany) supplemented with 10% (v/v) foetal calf serum, 50 μg streptomycin/mL and 50 units penicillin/mL. The cultures were incubated at 37°C in a humidified atmosphere containing 5% CO_2_ and the medium was replaced every 2 days after seeding. All experiments were approved by the Institutional Animal Care and Use Committee of Zhejiang University, Zhejiang Sheng, China.

### 2.2 Cells viability assay

Cell viability was determined using the 3‐(4,5‐dimethylthiazol‐2‐yl)‐2,5‐diphenyltetrazolium bromide (MTT) assay. Chondrocytes at passage 2 (5 × 10^3^ cells/well) were seeded in 96‐well plates and incubated with various concentrations of FSTL1 (Sigma Aldrich; Merck KGaA) for 24 hours. Then, 20 μL of MTT (5 mg/mL) was added to each well. After a 4‐h incubation, the culture medium was removed, an equal volume of DMSO was added to resuspend the formazan, and the absorbance at 570 nm was measured.

### 2.3 Quantitative real‐time polymerase chain reaction (RT‐PCR)

MMP‐1, MMP‐3, MMP‐13, cyclooxygenase (COX)‐2, inducible nitric oxide synthase (iNOS), IL‐1β, IL‐6 and tumour necrosis factor (TNF)‐α expression was analysed using an RT‐PCR system (Bio‐Rad, Marnes‐la‐Coquette, France). TRIzol reagent (Thermo Fisher Scientific, Inc, Waltham, MA, USA) was used according to the manufacturer's instructions to extract the total RNA of chondrocytes incubated with different concentrations of FSTL1 for 24 hours in 6‐well plates. For the investigated genes, mRNA expression was normalized to that of GAPDH. The primers, amplicon length and annealing temperatures are described in Table 1. Relative gene expression was calculated using the 2^–∆∆Ct ^method.

**Table 1:** Real‐time PCR primers and conditions

Gene	Genbank accession	Primer sequences (5'‐3')	Size (bp)	Annealing (℃)
GAPDH	NM_017008.4	GAAGGTCGGTGTGAACGGATTTG	127	60
		CATGTAGACCATGTAGTTGAGGTCA		
MMP‐1	NM_001134530.1	GCTTAGCCTTCCTTTGCTGTTGC	136	60
		GACGTCTTCACCCAAGTTGTAGTAG		
MMP‐3	NM_133523	CTGGGCTATCCGAGGTCATG	77	60
		TGGACGGTTTCAGGGAGGC		
MMP‐13	NM_133530	CAACCCTGTTTACCTACCCACTTAT	85	60
		CTATGTCTGCCTTAGCTCCTGTC		
COX‐2	S67722	CGCATTCTTTGCCCAGCACTTCACT	190	60
		CACCTCTCCACCGATGACCTGATA		
iNOS	NM_012611.3	GCTCGGGCTGAAGTGGTATGC	127	60
		GAAGTCTCGGACTCCAATCTCGGT		
IL‐1β	NM_031512	CCTAGGAAACAGCAATGGTCGGGAC	239	60
		GTCAGAGGCAGGGAGGGAAACAC		
IL‐6	NM_012589	CGCAAGAGACTTCCAGCCAG	146	60
		GCCTCCGACTTGTGAAGTGGT		
TNF‐α	NM_012675	GACCCCTTTATCGTCTACTCCTC	144	60
		GCCACTACTTCAGCGTCTCGT		

### 2.4 Enzyme‐linked immunosorbent assay (ELISA)

Commercially available ELISA kits were used to quantify the levels of IL‐1β, IL‐6 and TNF‐α in the medium (R&D Systems, Minneapolis, MN, USA) according to the manufacturer's guidelines.

### 2.5 Western blotting

The chondrocytes were washed with cold phosphate‐buffered saline and lysed using cell lysis buffer (Cell Signaling Technology, Danvers, MA, USA). Cell proteins were separated by sodium dodecyl sulphate‐polyacrylamide gel electrophoresis on 10% polyacrylamide gels and then transferred onto membranes. After the membranes had been blocked with 5% bovine serum albumin for 1 hour at room temperature, the proteins were probed overnight at 4°C using primary antibodies against, MMP‐1, MMP‐3, MMP‐13, COX‐2, iNOS, NF‐κB p65, p‐NF‐κB p65 and p‐IκBα (Cell Signaling Technology). The membranes were then washed with TBST and incubated with secondary antibodies for 1 hour at room temperature. The reacted protein bands were detected using an enhanced chemiluminescence substrate (Fude Biological Technology, Hangzhou, China) and exposure to X‐Omat film (Kodak, Rochester, NY, USA) according to the manufacturer's protocol. The relative amount of proteins was quantitated with Quantity One software (Bio‐Rad) and normalized to GAPDH.

### 2.6 Statistical analysis

All experiments were performed in triplicate using independent samples. The results are expressed as means ± SD and were analysed by one‐way analysis of variance. Comparison between the groups was performed with post hoc Tukey's test. Statistical analyses were performed using SPSS for Windows software (version 19.0; IBM Corp., Armonk, NY, USA). A *P *< 0.05 was considered to indicate statistical significance.

## 3 RESULTS

### 3.1 Effects of FSTL1 on cells viability

**Figure 1:**
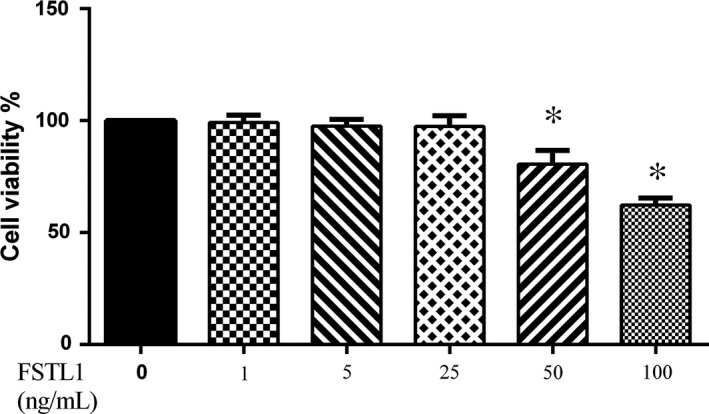
Effects of follistatin‐like protein 1 (FSTL1) on cell viability. Chondrocytes were treated with 1, 5, 25, 50 or 100 ng FSTL1/mL for 24 h. Cells incubated without FSTL1 were used as controls. Each column represents the mean ± SD. *P < 0.05 vs control group (FSTL1: 0 ng/mL)

As shown in Figure 1, FSTL1 at a concentration of 0‐25 ng/mL was not cytotoxic for the cultured chondrocytes. At FSTL1 concentrations of 50 and 100 ng/mL, cell viability decreased remarkably compared to non‐treated cells. Therefore, in this study, FSTL1 was used at a dose of 0‐25 ng/mL.

### 3.2 Effects of FSTL1 on MMP‐1, MMP‐3, MMP‐13, INOS and COX‐2 expression

**Figure 2:**
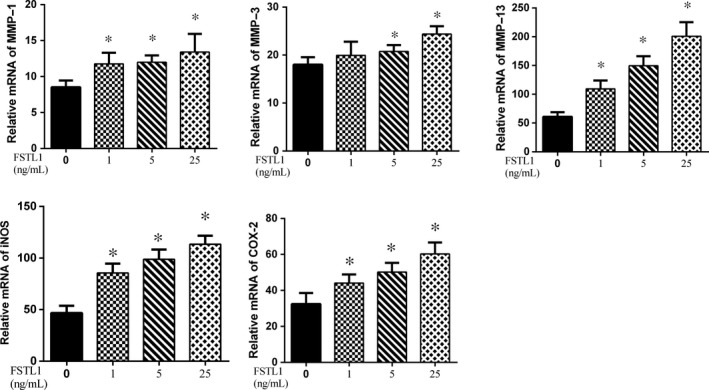
Effects of follistatin‐like protein 1 on the expression of matrix metalloproteinase (MMP)‐1, MMP‐3, MMP‐13, inducible nitric oxide synthase (iNOS) and cyclooxygenase (COX)‐2 mRNA. Chondrocytes were pre‐treated with 1, 5 or 25 ng FSTL1/mL for 24 h. Each column represents the mean ± SD. All three concentrations strongly increased MMP‐1, MMP‐13, iNOS and COX‐2 mRNA levels. MMP‐3 mRNA expression increased only in response to the higher doses of FSTL1 (5 and 25 ng/mL). *P < 0.05 vs control group

**Figure 3:**
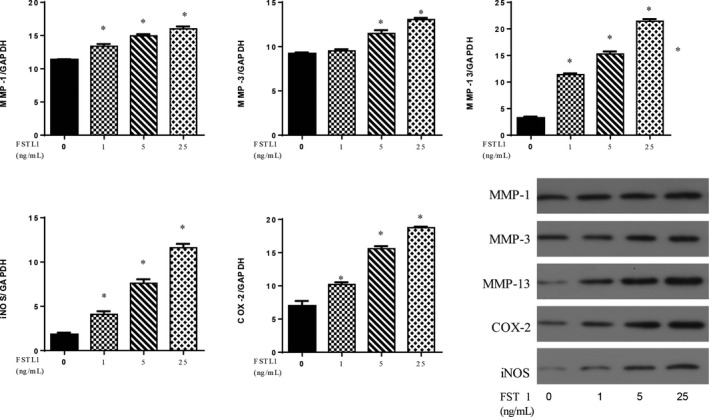
Effects of FSTL1 on MMP‐1, MMP‐3, MMP‐13, iNOS and COX‐2 protein expression. Each column represents the mean ± SD. FSTL1 (1, 5 and 25 ng/mL) increased MMP‐1, MMP‐13 iNOS and COX‐2 protein expression (*P* < 0.05), consistent with the RT‐PCR results. A low dose of FSTL1 (1 ng/mL) had no effect on MMP‐3 protein levels. *P < 0.05 vs control group

The effects of FSTL1 on MMP‐1, MMP‐3, MMP‐13, iNOS and COX‐2 mRNA expression were assessed using RT‐PCR (Figure 2). The results showed that FSTL1 significantly increased the expression of MMP‐1, MMP‐13 iNOS and COX‐2 (*P* < 0.05) compared to the untreated group. However, a low dose of FSTL1 (1 ng/mL) had no effect on MMP‐3 expression. Western blot (Figure 3) revealed the increased expression of MMP‐1, MMP‐13 iNOS and COX‐2 (*P* < 0.05) in cells treated with FSTL1, consistent with the quantitative RT‐PCR results. There was no significant change in the expression of MMP‐3 protein after FSTL1 stimulation (1 ng/mL).

### 3.3 Effects of FSTL1 on IL‐1β, TNF‐α and IL‐6 production

**Figure 4:**
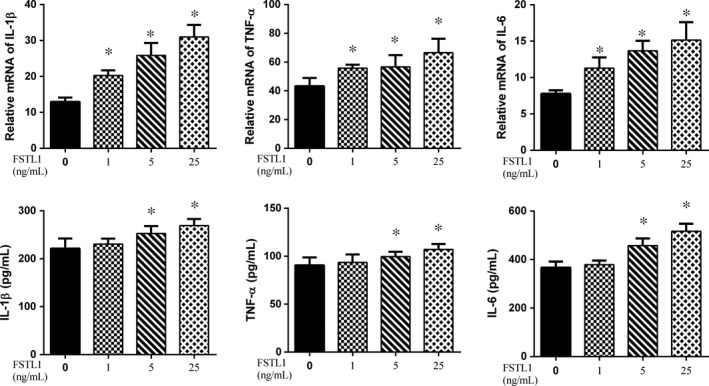
Effects of FSTL1 on IL‐1β, TNF‐α and IL‐6 expression in chondrocytes. Each column represents the mean ± SD. FSTL1 (1, 5 and 25 ng/mL) significantly induced IL‐1β, TNF‐α and IL‐6 mRNA levels, as determined by RT‐PCR. Only the high dose (5 and 25 ng/mL) of FSTL1‐stimulated IL‐1β, TNF‐α and IL‐6 protein expression, as determined by ELISA. *P < 0.05 vs control group

The levels of IL‐1β, TNF‐α and IL‐6 expression in FSTL1‐treated chondrocytes were determined by RT‐PCR and ELISA (Figure 4). A significant up‐regulation of these pro‐inflammatory mediators in cells incubated with 5 and 25 ng FSTL1/mL, at both gene and protein levels, was observed, especially in the high‐dose FSTL1 group. In cells treated with 1 ng FSTL1/mL, only the mRNA levels of IL‐1β, TNF‐α and IL‐6 were increased.

### 3.4 Effects of FSTL1 on NF‐κB activation in chondrocytes

**Figure 5:**
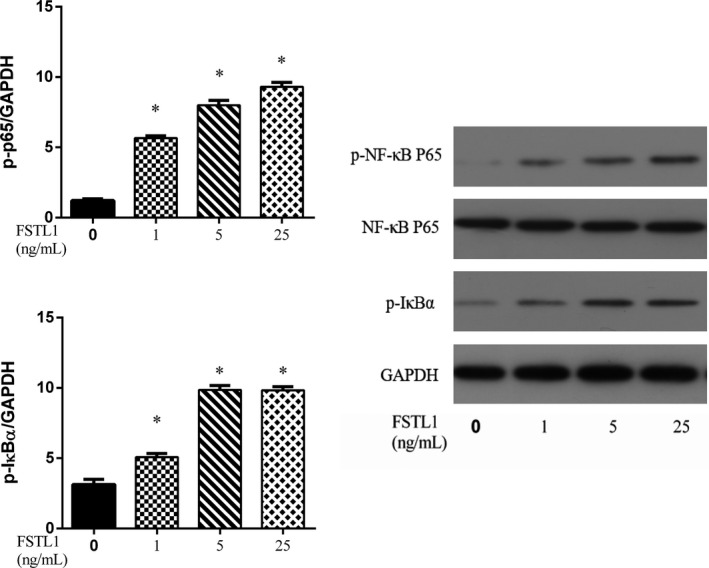
Effects of FSTL1 on nuclear factor kappa B (NF‐κB) activation in chondrocytes. FSTL1 (1, 5 and 25 ng/mL) induced the degradation of IκBα by simulating its phosphorylation. FSTL1 also significantly increased the level of p‐p65. GAPDH was used as the endogenous control. *P < 0.05 vs control group

The potential mechanism underlying the observed effects of FSTL1 was explored by examining the effect of FSTL1 on the NF‐κB signalling pathway (Figure 5). Western blotting showed that, compared to the control group, the expression of p‐NF‐κB p65 and phosphorylation of IκBα were significantly enhanced in the FSTL1‐treated group.

## 4 DISCUSSION

Follistatin‐like protein 1 (FSTL1) is a secreted extracellular glycoprotein involved in musculoskeletal system functions. In patients with intervertebral disc degeneration, serum FSTL1 levels were significantly higher than in the control group. In nucleus pulposus cells, FSTL1 induces the expression of the inflammatory mediators TNF‐α, IL‐1β, IL‐6, COX‐2 and MMP‐13, by activating both MAPK and NF‐κB pathways.^16^ A recent study showed that FSTL1 plays an important role in cartilage repair, by regulating chondrocyte proliferation and differentiation.^17^ However, as the only active cells in articular cartilage, chondrocytes are the primary target of extracellular matrix degradation.^18^ Therefore, in this study, we explored the direct relationship between chondrocytes and FSTL1. The results suggested that FSTL1 exacerbates OA by increasing the secretion of inflammatory and catabolic factors via the NF‐κB pathway.

Osteoarthritis is a chronic disease of the whole joint characterized by inflammation involving the cartilage, synovium, joint capsule, subchondral bone and meniscus.^19^ IL‐1β and TNF‐α promote the production of MMPs and are thus key inflammatory mediators in OA progression.^20^ However, both cytokines also inhibit the synthesis of extracellular matrix proteins.^21^ Cho et al showed that TNF‐α induces apoptosis in chondrocytes, by increasing apoptosis‐related gene expression.^22^ Thus, an imbalance between extracellular matrix (ECM) catabolism and anabolism may lead to the loss of articular cartilage. IL‐6 exacerbates OA progression by inhibiting the synthesis of type II collagen, the main component of the ECM.^23^ In OA patients, an increased circulating level and over‐expression in synovial fluids of IL‐6 have been described.^24^ A previous study implicated FSTL1 in the pathogenesis of reactive arthritis, RA and lumbar disc herniation. Interactions between FSTL1 and pro‐inflammatory cytokines (TNF‐α, IL‐1β and IL‐6) were demonstrated in fibroblast‐like synoviocytes. Su et al reported that FSTL1 promotes the expressions of TNF‐α, IL‐1β and IL‐6 in the cultured fibroblast‐like synoviocytes of OA patients.^25^ Our study showed increased expression of TNF‐α, IL‐1β and IL‐6 in FSTL1‐treated chondrocytes.

Inducible nitric oxide synthase (iNOS) and COX‐2 play pivotal roles in many inflammatory diseases including OA. Previous studies showed that excessive production of oxidants such as nitric oxide (NO) has been linked with the apoptosis of chondrocytes and synoviocytes.^26^ 15708893 nitric oxide is demonstrated to induce the expressions of MMPs in articular chondrocytes^27^ 7529496. Moreover, inflammatory cytokines such as IL‐1 and TNF‐α can directly increase the gene expression of iNOS.^28^ 1658153. It was suggested that it may provide beneficial chondroprotective effects in the pathophysiological structural changes of OA and reduce the activation of MMPs by using a selective inhibitor of iNOS.^29^ 9663486. Over‐expression of COX‐2 results in excessive production of PGE2, which is a crucial mediator in the degradation of both aggrecan and type II collagen.^30^ 18802112.The selective COX‐2 inhibitors, such as celecoxib and meloxicam, are widely used on the market to relieve pain and inflammation in OA. In our study, FSTL1 also significantly promotes the expression of iNOS and COX‐2. Taken together, these results suggest that FSTL1 exerts its pro‐inflammatory function by interacting with the joint tissue and increasing the secretion of pro‐inflammatory cytokines.

Matrix metalloproteinases (MMPs) are a family of proteases involved in ECM degradation, catabolism and turnover. The collagenase MMP‐1 is responsible for the digestion of interstitial collagens,^31^ MMP‐3 degrades proteoglycans and activates procollagenase,^32^ and MMP‐13, an extracellular‐matrix‐degrading enzyme, promotes the degradation of proteoglycans and activates other collagenases.^33^ Previous reports showed that FSTL1 stimulate the expression of MMPs in synoviocytes and nucleus pulposus cells.^14^, ^16^ Therefore, in this study, we investigated whether FSTL1 affects the expression of MMPs in chondrocytes. Our results showed that FSTL1 significantly increased the expression of MMP‐1 and MMP‐13 in a dose‐dependent manner.

The transcription factor NF‐κB regulates the expression of a large number of inflammatory genes, as well as MMP production. In the cytosol, NF‐κB protein remains in an inactive state as long as it is bound to its inhibitor, IκBα.^34^ In response to chemical signals or mechanical stress, the IκBα unit is phosphorylated and degraded, resulting in the nuclear translocation of NF‐κB and the induction of gene transcription.^35^ Therefore, NF‐κB expression in chondrocytes may be a valuable target in the prevention and treatment of OA.^36^ Previous studies showed that FSTL1 stimulates NF‐κB signalling.^37^, ^38^ The results of our Western blot analysis indicated that FSTL1 induces the activation of NF‐κB p65 by promoting IκB‐α degradation. These findings suggest that the pro‐inflammatory effects of FSTL1 in chondrocytes are mediated by its activation of NF‐κB signalling.

A limitation of our study is that only in vitro model, monolayer cultured chondrocytes, is applied to mimic the development process of OA and evaluate the impact of pro‐inflammatory factor FSTL1 on OA. Further research, such as 3D cultivation and in vivo animal OA model, should be carried out to evidence its phenotype and mechanism.

In conclusion, our study demonstrated that FSTL1 significantly increases the mRNA and protein levels of MMP‐1, MMP‐13, COX‐2, iNOS, TNF‐α, IL‐1β and IL‐6 in chondrocytes. This pro‐inflammatory effect can be attributed to an increase in NF‐κB activity. FSTL1 may therefore be a therapeutic target in OA.

## CONFLICT OF INTEREST

The authors confirm that there are no conflicts of interest.

## References

[1] Dieppe PA , Lohmander LS . Pathogenesis and management of pain in osteoarthritis. Lancet. 2005;365:965‐973. 15766999 10.1016/S0140-6736(05)71086-2

[2] Taruc‐Uy RL , Lynch SA . Diagnosis and treatment of osteoarthritis. Prim Care. 2013;40:821‐836, vii. 24209720 10.1016/j.pop.2013.08.003

[3] Ikeuchi M , Kamimoto Y , Izumi M , et al. Effects of dexamethasone on local infiltration analgesia in total knee arthroplasty: a randomized controlled trial. Knee Surg Sports Traumatol Arthrosc. 2014;22:1638‐1643. 23306715 10.1007/s00167-013-2367-5

[4] Steinecker‐Frohnwieser B , Weigl L , Kullich W , Lohberger B . The disease modifying osteoarthritis drug diacerein is able to antagonize pro inflammatory state of chondrocytes under mild mechanical stimuli. Osteoarthritis Cartilage. 2014;22:1044‐1052. 24857974 10.1016/j.joca.2014.05.008

[5] Lane NE , Schnitzer TJ , Birbara CA , et al. Tanezumab for the treatment of pain from osteoarthritis of the knee. N Engl J Med. 2010;363:1521‐1531. 20942668 10.1056/NEJMoa0901510 PMC6896791

[6] Shibanuma M , Mashimo J , Mita A , et al. Cloning from a mouse osteoblastic cell line of a set of transforming‐growth‐factor‐beta 1‐regulated genes, one of which seems to encode a follistatin‐related polypeptide. Eur J Biochem. 1993;217:13‐19. 7901004 10.1111/j.1432-1033.1993.tb18212.x

[7] Zhang Y , Xu X , Yang Y , et al. Deficiency of follistatin‐like protein 1 accelerates the growth of breast cancer cells at lung metastatic sites. J Breast Cancer. 2018;21:267‐276. 30275855 10.4048/jbc.2018.21.e43 PMC6158165

[8] Jin YK , Li XH , Wang W , et al. Follistatin‐like 1 promotes bleomycin‐induced pulmonary fibrosis through the transforming growth factor beta 1/mitogen‐activated protein kinase signaling pathway. Chin Med J. 2018;131:1917‐1925. 30082522 10.4103/0366-6999.238151 PMC6085847

[9] Xiao Y , Zhang Y , Chen Y , et al. Inhibition of microRNA‐9‐5p protects against cardiac remodeling following myocardial infarction in mice. Hum Gene Ther. 2018; [Epub ahead of print]. 10.1089/hum.2018.059 30101604

[10] Chaly Y , Marinov AD , Oxburgh L , et al. FSTL1 promotes arthritis in mice by enhancing inflammatory cytokine/chemokine expression. Arthritis Rheum. 2012;64:1082‐1088. 22006268 10.1002/art.33422 PMC3276726

[11] Wilson DC , Marinov AD , Blair HC , et al. Follistatin‐like protein 1 is a mesenchyme‐derived inflammatory protein and may represent a biomarker for systemic‐onset juvenile rheumatoid arthritis. Arthritis Rheum. 2010;62:2510‐2516. 20506332 10.1002/art.27485 PMC2921021

[12] Tanaka M , Ozaki S , Osakada F , et al. Cloning of follistatin‐related protein as a novel autoantigen in systemic rheumatic diseases. Int Immunol. 1998;10:1305‐1314. 9786430 10.1093/intimm/10.9.1305

[13] Tanaka M , Ozaki S , Kawabata D , et al. Potential preventive effects of follistatin‐related protein/TSC‐36 on joint destruction and antagonistic modulation of its autoantibodies in rheumatoid arthritis. Int Immunol. 2003;15:71‐77. 12502727 10.1093/intimm/dxg005

[14] Ni S , Li C , Xu N , et al. Follistatin‐like protein 1 induction of matrix metalloproteinase 1, 3 and 13 gene expression in rheumatoid arthritis synoviocytes requires MAPK, JAK/STAT3 and NF‐kappaB pathways. J Cell Physiol. 2018;234:454‐463. 29932210 10.1002/jcp.26580

[15] Wang Y , Li D , Xu N , et al. Follistatin‐like protein 1: a serum biochemical marker reflecting the severity of joint damage in patients with osteoarthritis. Arthritis Res Ther. 2011;13:R193. 22117761 10.1186/ar3522 PMC3334643

[16] Liu Y , Wei J , Zhao Y , et al. Follistatin‐like protein 1 promotes inflammatory reactions in nucleus pulposus cells by interacting with the MAPK and NFkappaB signaling pathways. Oncotarget. 2017;8:43023‐43034. 28498809 10.18632/oncotarget.17400 PMC5522124

[17] Chaly Y , Blair HC , Smith SM , et al. Follistatin‐like protein 1 regulates chondrocyte proliferation and chondrogenic differentiation of mesenchymal stem cells. Ann Rheum Dis. 2015;74:1467‐1473. 24641944 10.1136/annrheumdis-2013-204822

[18] Hwang HS , Kim HA . Chondrocyte apoptosis in the pathogenesis of osteoarthritis. Int J Mol Sci. 2015;16:26035‐26054. 26528972 10.3390/ijms161125943 PMC4661802

[19] Goldring MB , Otero M . Inflammation in osteoarthritis. Curr Opin Rheumatol. 2011;23:471‐478. 21788902 10.1097/BOR.0b013e328349c2b1 PMC3937875

[20] Kobayashi M , Squires GR , Mousa A , et al. Role of interleukin‐1 and tumor necrosis factor alpha in matrix degradation of human osteoarthritic cartilage. Arthritis Rheum. 2005;52:128‐135. 15641080 10.1002/art.20776

[21] Malemud CJ , Islam N , Haqqi TM . Pathophysiological mechanisms in osteoarthritis lead to novel therapeutic strategies. Cells Tissues Organs. 2003;174:34‐48. 12784040 10.1159/000070573

[22] Cho TJ , Lehmann W , Edgar C , et al. Tumor necrosis factor alpha activation of the apoptotic cascade in murine articular chondrocytes is associated with the induction of metalloproteinases and specific pro‐resorptive factors. Arthritis Rheum. 2003;48:2845‐2854. 14558090 10.1002/art.11390

[23] Poree B , Kypriotou M , Chadjichristos C , et al. Interleukin‐6 (IL‐6) and/or soluble IL‐6 receptor down‐regulation of human type II collagen gene expression in articular chondrocytes requires a decrease of Sp1.Sp3 ratio and of the binding activity of both factors to the COL2A1 promoter. J Biol Chem. 2008;283:4850‐4865. 18065760 10.1074/jbc.M706387200

[24] Livshits G , Zhai G , Hart DJ , et al. Interleukin‐6 is a significant predictor of radiographic knee osteoarthritis: the Chingford study. Arthritis Rheum. 2009;60:2037‐2045. 19565477 10.1002/art.24598 PMC2841820

[25] Ni S , Miao K , Zhou X , et al. The involvement of follistatin‐like protein 1 in osteoarthritis by elevating NF‐kappaB‐mediated inflammatory cytokines and enhancing fibroblast like synoviocyte proliferation. Arthritis Res Ther. 2015;17:91. 25888873 10.1186/s13075-015-0605-6 PMC4407312

[26] Maneiro E , Lopez‐Armada MJ , de Andres MC , et al. Effect of nitric oxide on mitochondrial respiratory activity of human articular chondrocytes. Ann Rheum Dis. 2005;64:388‐395. 15708893 10.1136/ard.2004.022152 PMC1755391

[27] Murrell GA , Jang D , Williams RJ . Nitric oxide activates metalloprotease enzymes in articular cartilage. Biochem Biophys Res Comm. 1995;206:15‐21. 7529496 10.1006/bbrc.1995.1003

[28] Stadler J , Stefanovic‐Racic M , Billiar TR , et al. Articular chondrocytes synthesize nitric oxide in response to cytokines and lipopolysaccharide. J Immunol. 1991;147:3915‐3920. 1658153

[29] Pelletier JP , Jovanovic D , Fernandes JC , et al. Reduced progression of experimental osteoarthritis in vivo by selective inhibition of inducible nitric oxide synthase. Arthritis Rheum. 1998;41:1275‐1286. 9663486 10.1002/1529-0131(199807)41:7<1275::AID-ART19>3.0.CO;2-T

[30] Attur M , Al‐Mussawir HE , Patel J , et al. Prostaglandin E2 exerts catabolic effects in osteoarthritis cartilage: evidence for signaling via the EP4 receptor. J Immunol. 2008;181:5082‐5088. 18802112 10.4049/jimmunol.181.7.5082

[31] Pardo A , Selman M . MMP‐1: the elder of the family. Int J Biochem Cell Biol. 2005;37:283‐288. 15474975 10.1016/j.biocel.2004.06.017

[32] Lin PM , Chen CT , Torzilli PA . Increased stromelysin‐1 (MMP‐3), proteoglycan degradation (3B3‐ and 7D4) and collagen damage in cyclically load‐injured articular cartilage. Osteoarthritis Cartilage. 2004;12:485‐496. 15135145 10.1016/j.joca.2004.02.012

[33] Li H , Wang D , Yuan Y , Min J . New insights on the MMP‐13 regulatory network in the pathogenesis of early osteoarthritis. Arthritis Res Ther. 2017;19:248. 29126436 10.1186/s13075-017-1454-2 PMC5681770

[34] Pahl HL . Activators and target genes of Rel/NF‐kappaB transcription factors. Oncogene. 1999;18:6853‐6866. 10602461 10.1038/sj.onc.1203239

[35] Napetschnig J , Wu H . Molecular basis of NF‐kappaB signaling. Ann Rev Biophys. 2013;42:443‐468. 23495970 10.1146/annurev-biophys-083012-130338 PMC3678348

[36] Roman‐Blas JA , Jimenez SA . NF‐kappaB as a potential therapeutic target in osteoarthritis and rheumatoid arthritis. Osteoarthritis Cartilage. 2006;14:839‐848. 16730463 10.1016/j.joca.2006.04.008

[37] Lau MC , Ng KY , Wong TL , et al. FSTL1 promotes metastasis and chemoresistance in esophageal squamous cell carcinoma through NFkappaB‐BMP signaling cross‐talk. Cancer Res. 2017;77:5886‐5899. 28883005 10.1158/0008-5472.CAN-17-1411

[38] Kim HJ , Kang WY , Seong SJ , et al. Follistatin‐like 1 promotes osteoclast formation via RANKL‐mediated NF‐kappaB activation and M‐CSF‐induced precursor proliferation. Cell Signal. 2016;28:1137‐1144. 27234130 10.1016/j.cellsig.2016.05.018

